# Supplementary material for: The Identification of Dual T-Cell and B-Cell Epitopes Within Viral Proteins Utilizing a Comprehensive Peptide Array Approach
Source: Vaccines (Basel). 2025 Feb 26;13(3):239. doi: 10.3390/vaccines13030239 (PMC11946625; doi:10.3390/vaccines13030239)

Supplementary Figures for "Identification of Dual T-Cell and B-Cell Epitopes within Viral Proteins Utilizing a Comprehensive Peptide Array Approach"

Figure S1. Immunoreactivity model architecture.

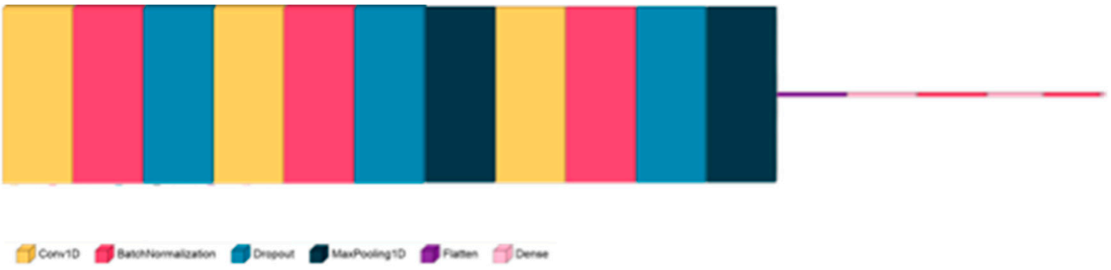

Figure S2. Antibody titers of PPV immunized mouse. P1 and P2 were PPV infected mouse, while N1, N2 and N3 were negative controls. Antibody titers were detected with PPV-coated ELISA. The OD value was detected at wavelength 450nM.

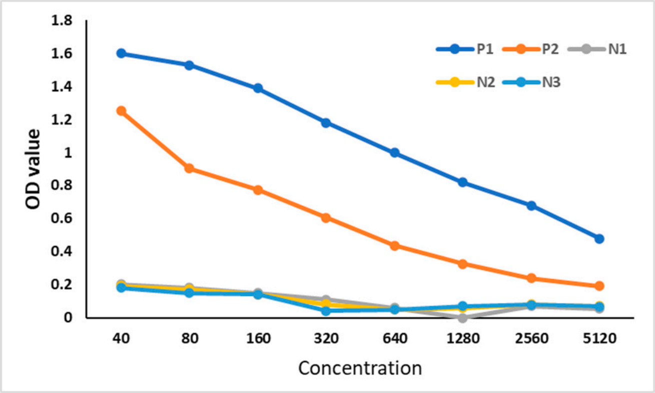

Figure S3. Performance of antibody reactivity model for each sample. Mouse with P.* as IDs are PPV infected, and others are negative controls. ρ is the Pearson correlation coefficient. All these models are trained on peptide array data with 60% of all peptides as training set, 20% as validation set and the left 20% as testing set.

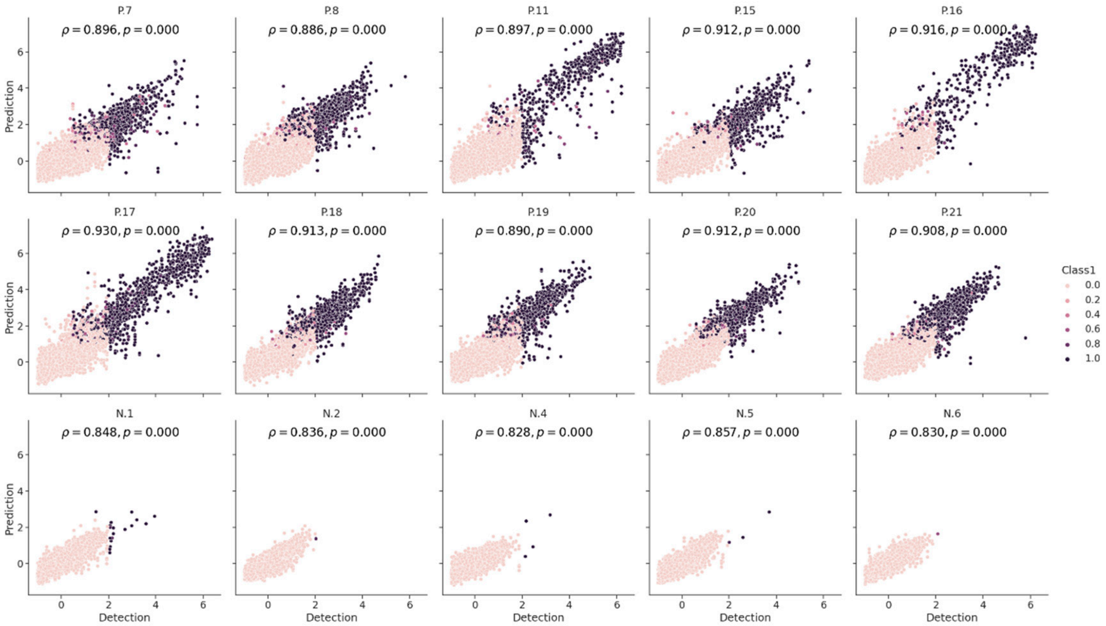

Figure S4. Raw experiment results of ELIspot and ELISA of mix peptides. **A.** Peptide-specific antibody detected after Boost1. The positive fraction to each peptide were shown in the right row. **B.** Peptide-specific antibody detected after Boost2. The positive fraction to each peptide were shown in the right row. **C**. IFN-γ-secreting T lymphocyte responses to peptides for each mouse after Boost1. Each spots represent a single T cell secreting γ-IFN specific for peptides P1-P14. IDs with P* are peptides and M* are mouse.

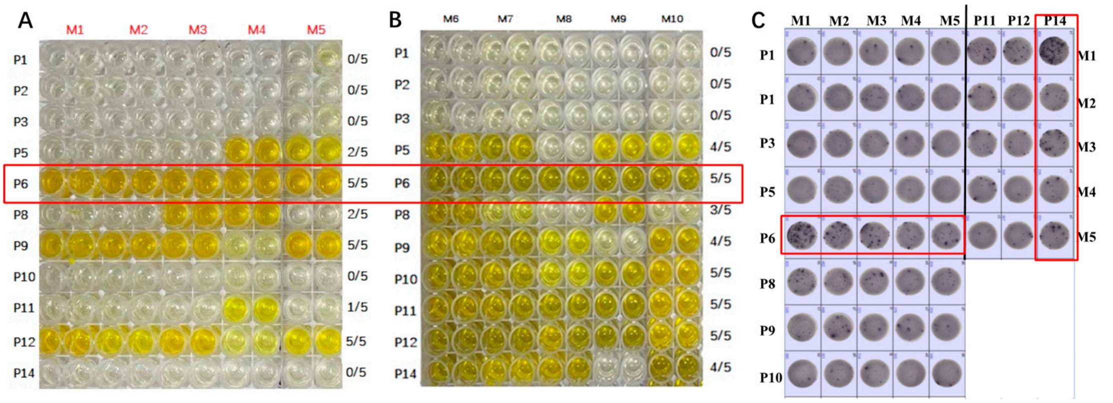

Figure S5. Performance of immunoreactivity model for P6 immunized mice. Mice with PPV6 as IDs are P6 peptide immunized, and others are negative controls. ρ is the Pearson correlation coefficient. All these models are trained on peptide array data with 60% of all peptides as training set, 20% as validation set and the left 20% as testing set.

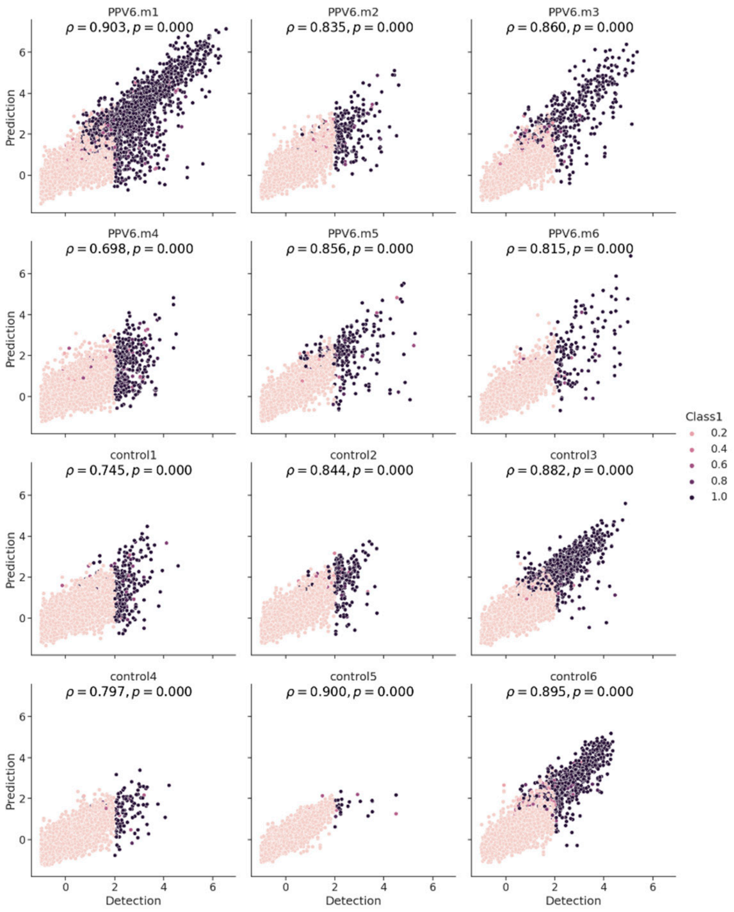

Figure S6. Possible important residuals explaining the efficiency of P6-3. A. With the high-signal peptide probes of each sample, we calculated the Zscore to define the enrichement of space-kmers by comparing to a background distribution of them. Then we also used the immunoreactivity model to predict reactivity of each space-kmer. All the enriched space-kmers were shown in scatterplot. Space-kmers with reactivity >=3 and included in P6-3 peptide or P6-4 peptide were colored red and blue respectively. **B.** Antigenicity was evaluated with Kolaskar-Tongaonkar method from IEDB. The P6-3 region formed a high peak and was colored red.

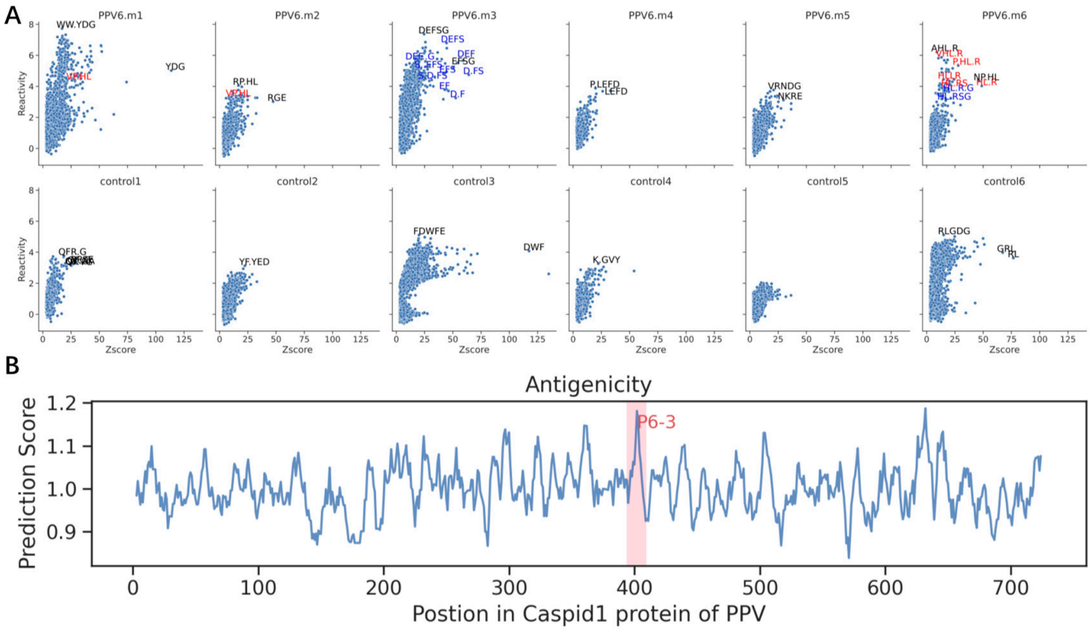

Supplement: Supplementary file 1 [file vaccines-13-00239-s001.zip › Supplementary figure.pdf]
